# Del Nido Cardioplegia can be safely administered in high-risk coronary artery bypass grafting surgery after acute myocardial infarction: a propensity matched comparison

**DOI:** 10.1186/s13019-014-0141-5

**Published:** 2014-10-30

**Authors:** Halit Yerebakan, Robert A Sorabella, Marc Najjar, Estibaliz Castillero, Linda Mongero, James Beck, Maliha Hossain, Hiroo Takayama, Mathew R Williams, Yoshifumi Naka, Michael Argenziano, Emile Bacha, Craig R Smith, Isaac George

**Affiliations:** Department of Surgery, Division of Cardiothoracic Surgery, College of Physicians and Surgeons of Columbia University, New York Presbyterian Hospital, MHB 7GN-435, 177 Fort Washington Ave, New York, 10032 NY USA

**Keywords:** Coronary artery bypass grafting, Myocardial protection, Acute myocardial infarction

## Abstract

**Objective:**

Del Nido (DN) cardioplegia solution provides a depolarized hyperkalemic arrest lasting up to 60 minutes, and the addition of lidocaine may limit intracellular calcium influx. Single-dose DN cardioplegia solution may offer an alternative myocardial protection strategy to multi-dose cold whole blood (WB) cardioplegia following acute myocardial infarction (AMI).

**Methods:**

We retrospectively reviewed 88 consecutive patients with AMI undergoing coronary artery bypass (CABG) surgery with cardioplegic arrest between June 2010 to June 2012. Patients exclusively received WB (n = 40, June 2010-July 2011) or DN (n = 48, August 2011-June 2012) cardioplegia. Preoperative and postoperative data were retrospectively reviewed and compared using propensity scoring.

**Results:**

No significant difference in age, maximum preoperative serum troponin level, ejection fraction, and STS score was present between DN and WB. A single cardioplegia dose was given in 41 DN vs. 0 WB patients (p < 0.001), and retrograde cardioplegia was used 10 DN vs. 31 WB patients (p < 0.001). Mean cardiopulmonary bypass and cross clamp times were significantly shorter in the DN group versus WB group. Tranfusion rate, length of stay, intra-aortic balloon pump requirement, post-operative inotropic support, and 30-day mortality was no different between groups. One patient in the WB group required a mechanical support due to profound cardiogenic shock.

**Conclusions:**

DN cardioplegia may provide equivalent myocardial protection to existing cardioplegia without negative inotropic effects in the setting of acute myocardial infarction.

**Electronic supplementary material:**

The online version of this article (doi:10.1186/s13019-014-0141-5) contains supplementary material, which is available to authorized users.

## Background

Despite advances in surgical technique and patient selection, mortality after coronary artery bypass grafting surgery (CABG) for acute myocardial infarction (AMI) remains high at 4-10% [1],[2]. Myocardial protection in this setting is complicated by subsequent ischemia-reperfusion injury, oxidative stress and intracellular Ca^2+^ overload [3], all of which may contribute to post-operative myocardial dsyfunction. Current cardioplegia options include whole blood cardioplegia (WB), Buckberg solution, and warm blood cardioplegia. However, conflicting evidence exists regarding the superiority of one solution compared to another. Moreover, solutions designed specifically to address metabolic changes after AMI or cardiogenic shock, such as Buckberg solution [4], can be cumbersome to deliver and are not universally adopted.

Del Nido (DN) cardioplegia was formulated to act as single-dose administration in pediatric patients. Compared to the traditional 4:1 blood cardioplegia, DN is more dilute (1:4, blood:crystalloid), has lower Ca^+2^, and contains lidocaine (140 mg/L) (Table 1) (Compass-Baxter Healthcare Inc., Edison, NJ). In pediatric patients, DN cardioplegia has been shown to result in lower postoperative troponin release compared to WB cardioplegia [5]. However, its use in adults has not been studied, and its unknown effects on the complex derangements after AMI have led surgeons to question its use in this sick patient population. We sought to evaluate the clinical outcomes of DN cardioplegia in CABG after AMI compared to standard 4:1 WB solution.

**Table 1:** **Composition of Whole Blood (WB) and del Nido (DN) cardioplegia solutions**

	4:1 Blood: Cardiogplegia	Del Nido
Na (mmol/L)	136-152	143-153
K (mmol/L)	24	24
Cl (mmol/L)	126	132
Mg (mmol/L)	2	6.2
Ca (mmol/L)		0.4
Lidocaine (mg/L)	200 (before XC release)	140
Mannitol (g/L)	12.5	2.6
NaHCO3	50	26

## Methods

### Patient population

Between July 2010 and July 2012, a total of 828 consecutive patients underwent CABG with or without concomitant cardiac surgical procedures at our institution. Out of 828 patients, 88 consecutive patients with AMI undergoing isolated CABG surgery with cardioplegic arrest were identified. AMI was defined as topononin I > 1 ng/mL within one week prior to operation. Demographic and clinical outcome data were retrospectively collected from chart review, including age, sex, race, body mass index (BMI), and comorbid medical conditions, including diabetes, hypertension, chronic obstructive pulmonary disease, renal failure, smoking history, severe aortic wall calcification, peripheral arterial disease, cerebrovascular disease, atrial fibrillation, and prior AMI, percutaneous coronary intervention, or cardiac surgery. The preoperative clinical status, including Society of Thoracic Surgeons (STS) risk score for mortality, New York Heart Association (NYHA) symptom class, echocardiographic data, and requirement for intra-aortic balloon pump (IABP) before surgery, was documented. This study met all guidelines of the Institutional Review Board of Columbia University.

### Study design

In the current study, clinical outcomes before and after adoption of DN were compared using a propensity score matching analysis. The first group (n = 40) was composed of a 1-year cohort (July 2010-June 2011) of patients who underwent isolated CABG following AMI with exclusive use of WB cardioplegia for myocardial protection (WB group). The second group (n = 48) was a similar 1-year cohort (July 2011-July 2012) of patients who underwent isolated CABG following AMI with exclusive use of DN cardioplegia for myocardial protection (DN group). A propensity score matching, based on Greedy 5 to 1 digital matching algorithm [6], was used to reduce major patient characteristic differences between groups. Propensity score matching (1:1) identified 40 matched pairs (in each group) for analysis.

The primary outcomes included low output syndrome (LCOS) and in-hospital mortality. The secondary outcomes were duration of cross-clamp (XC) and cardiopulmonary bypass (CPB), volume of cardioplegia used, red blood cell transfusion rate, and in-hospital complications. LCOS was defined as, if the patient required an IABP or mechanical circulatory support (MCS) or extra-corporeal membrane oxygenation (ECMO) in the operating room in order to be weaned from CPB or in the intensive care unit because of hemodynamic compromise. LCOS was also diagnosed if the patient required inotropic medication (at least two of either vasopressin, dobutamine, milrinone, or epinephrine) to maintain systolic blood pressure. Patients who required a renal dose of dopamine (≤3 μg/kg) or a single inotropic medication support were not considered to have LCOS.

### Operative details, cardioplegia, and complications

All surgeries were performed using a standard general anesthesia protocol, median sternotomy approach, employing cardiopulmonary bypass with mild systemic hypothermia (30 to 34°C). Intraoperative transesophageal echocardiography was routinely employed. Myocardial protection was achieved with either WB or DN cardioplegia as follows. In both groups, the heart was arrested with an induction dose (1 liter) of cold (4°C) cardioplegia using antegrade and/or retrograde delivery (see Table 1 for details). In addition, repeated doses of WB cardioplegia was given via saphenous vein grafts or through the retrograde cannula at 20 min intervals in WB patients only. A second dose (500 ml) of DN was only given if the XC exceeded 90 minutes.

The operative details that were collected included priority of surgery (elective, urgent, emergent), cardio-pulmonary bypass time, global ischemic time, amount and method of cardioplegia, and number of transfusions. Postoperative in-hospital complications included need for IABP, ECMO, or MCS, inotrope dependence on intensive care unit admission, ventricular or atrial arrhythmia, need for permanent pace maker, respiratory failure, renal failure, sepsis or endocarditis, sternal wound infection, gastrointestinal bleeding, stroke, MI, reoperation for bleeding, and death before discharge.

### Propensity score matching and statistical analysis

To permit an independent comparison, logistic regression and the Greedy 5 to1 Digit Match macro was used to generate the propensity scores employed for matching [6]. The multivariable logistic regression was run to compute propensity scores for the two groups (WB and DN) based on the following covariates: age, gender, STS score, NYHA class, body mass index (BMI), diabetes, hypertension, hyperlipidemia, preoperative cardiogenic shock, congestive heart failure, preoperative left ventricular ejection fraction (LVEF), number of diseased vessels, renal failure, peripheral vascular disease, chronic obstructive pulmonary disease, and reoperative cardiac surgery. Each patient who had WB was matched to one patient who had DN cardioplegia with the closest propensity score.

Descriptive statistics were used to describe patient characteristics. Categorical data were represented as frequency distributions and percentages. Continuous variables were expressed as mean ± standard deviation (SD). Univariate analysis of continuous variables was performed using student *t*-tests, whereas categorical variables were compared using chi-square test of homogeneity and independence in contingency tables. All p-values were two-sided. Data were analyzed using IBM SPSS Statistics 21.0 (IBM Corp., Armonk, NY, USA).

## Results

### Baseline demographics

The mean age of the patients was 67.7 ± 12.6 years (range 28-93 years), 57.5% were male, and mean BMI was 28.2 ± 5.7 kg/m^2^. Mean maximum preoperative serum troponin level was 15.1 ng/ml (range 1-84.5 ng/ml). Mean STS risk score for mortality of all patients was 4.3% (range 0.4-31.1%). The baseline clinical characteristics of the 2 propensity-matched groups (WB vs. DN) were balanced in all measured characteristics and summarized in Table 2.

**Table 2:** **Baseline demographics**

	WB	DN	***p***
n	40	40	
Age (years)	66.6 ± 13.5	68.7 ± 11.7	0.472
Gender (male) (%)	21 (45.7)	25 (54.3)	0.366
BMI (kg/m^2^)	28.2 ± 5.9	28.1 ± 5.7	0.926
STS risk score (%)	4.6 ± 6.9	3.9 ± 4.2	0.545
Troponin I level (mean) (ng/ml)	15.6 ± 23	14.8 ± 21	0.915
NYHA class (%)			
1	5 (12.5)	6 (15)	0.921
2	22 (55)	21 (52.5)	
3	12 (30)	11 (27.5)	
4	1 (2.5)	2 (5)	
Diabetes (%)	18 (45)	22 (55)	0.371
Insulin-dependent (%)	8 (20)	7 (17.5)	0.775
Hypertension (%)	34 (85)	34 (85)	1
Congestive heart failure (%)	5 (12.5)	10 (25)	0.152
LVEF (%)	49.4 ± 12	42.4 ± 12	0.13
Mitral regurgitation > Grade 2 (%)	10 (25)	9 (22.5)	0.925
Tricuspid regurgitation > Grade 2 (%)	3 (7.5)	4 (10)	0.283
Triple-vessel disease (%)	22 (55)	30 (75)	0.061
Quadruple-vessel disease (%)	12 (22.5)	8 (22.5)	
Cardiogenic shock (%)	6 (15)	5 (12.5)	0.745
Pre-operative IABP support (%)	5 (12.5)	7 (17.5)	0.531
Previous cardiac surgery (%)	3 (7.5)	1 (2.5)	0.305
Preoperative arrhythmia (%)	10 (25)	6 (15)	0.264
Preoperative Atrial Fibrillation (%)	9 (22.5)	3 (7.5)	0.060
Preoperative stroke (%)	7 (17.5)	6 (15)	0.762
Peripheral vascular disease (%)	8 (20)	8 (20)	1
Preoperative renal failure (%)	4 (10)	6 (15)	0.499
Preoperative Dialysis (%)	1 (2.5)	0 (0)	0.314
COPD (%)	3 (7.5)	5 (12.5)	0.456
Cirrhosis (%)	1 (2.5)	1 (2.5)	1

Values are means ± SD, or counts (%). BMI-body mass index, STS-Society of Thoracic Surgeons, NYHA-New York Heart Association, LVEF-left ventricular ejection fraction, COPD-chronic obstructive pulmonary disorder.

### Operative data

**Figure 1:**
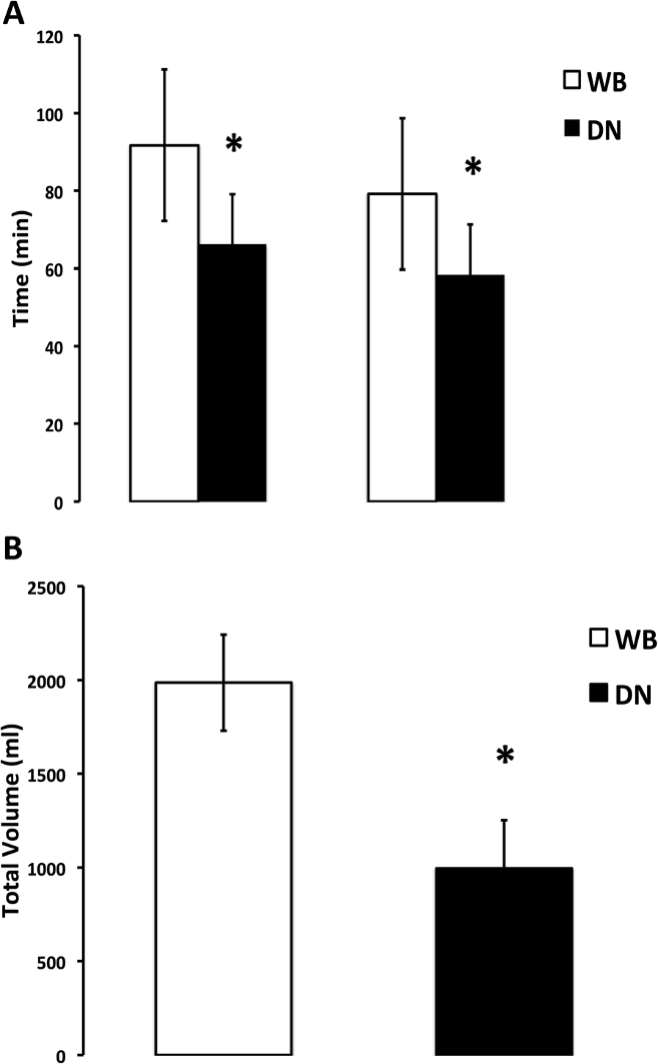
**Intraoperative variables. A)** Cardiopulmonary bypass and aortic cross clamp times between groups, **B)** Total cardioplegia volume given during operation by treatment group. (Abbreviations: CPB=cardiopulmonary bypass, DN=del Nido cardioplegia group, WB=whole blood cardioplegia group, XC=aortic cross-clamp).

Use of DN cardioplegia was associated with significantly shorter CPB and XC times (both, p < 0.001; Figure 1A). Additionally, administration of DN cardioplegia resulted in significant differences in manner and amount of delivered cardioplegia (all, p < 0.03, Figure 1B). An initial cold antegrade cardioplegia was used in all patients. A single cardioplegia dose was given in 33 DN vs. 5 WB patients (p < 0.001), and retrograde cardioplegia was used in only 8 DN vs. 31 WB patients (p < 0.001). Mean packed red blood cell (PRBC) transfusion requirements on CPB tended to be lower with DN versus WB during CBP (1.4 ± 1.5 vs. 0.9 ± 1.3, WB vs. DN, p = 0.113). The operative profiles of the 2 propensity-matched groups (WB vs. DN) are summarized in Table 3.

**Table 3:** **Operative profile (Matched Groups)**

	WB	DN	***p***
n	40	40	
***Intraoperative variables***			
Number of grafts	3.2 ± 0.7	3.3 ± 0.6	0.708
Lowest body temperature on CPB (°C)	32.3 ± 1	34.1 ± 2	<0.001
Cardioplegia Delivery (%)			
Antegrade	9 (22.5)	32 (80)	<0.001
Antegrade + Retrograde	31 (77.5)	8 (20)	<0.001
Repeated Dose (Ante or Retro)	35 (87.5)	7 (17.5)	<0.001
Cardioplegia Volume			
Total initial dose (ml)	1062 ± 198	954 ± 235	0.029
Antegrade-initial (ml)	786 ± 295	944 ± 235	0.010
Retrograde-initial (ml)	266 ± 221	11 ± 47	<0.001
Number of additional doses	3.2 ± 2	0.2 ± 0.4	<0.001
Additional dose amount (ml)	926 ± 591	42 ± 97	<0.001
Reinstitute CPB (%)	1 (2.5)	0 (0)	0.314
PRBC transfusion during CPB	1.4 ± 1.5	0.9 ± 1.3	0.113

Values are means ± SD, or counts (%). CPB-cardioplumonary bypass, PRBC-packed red blood cells.

### Postoperative outcomes

**Figure 2:**
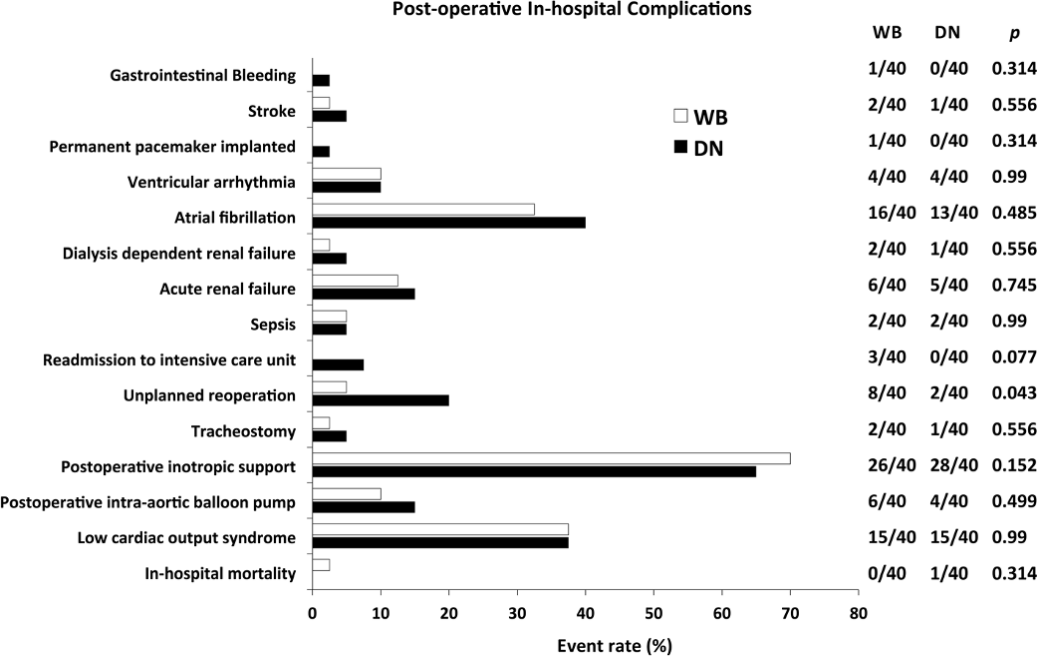
**In-hospital Complications by type of cardioplegia technique.** Observed in-hospital post-operative complication rates in patients underdoing coronary artery bypass grafting after acute myocardial infarction stratified by cardioplegia technique with number of patients (n) represented next to event rate of each variable for whole blood and Del Nido, respectively. No significant differences were found for any subgroup except unplanned re-operation. The p values of each variable are presented next to the number of events.

Postoperative outcome details are highlighted in Figure 2 and Table 4. In-hospital mortality was statistically comparable between groups (p = 0.314). One patient (2.5%) in DN group died due to profound cardiogenic shock and there was no hospital mortality in WB group. Additionally, the prevalence of LCOS was identical in the 2 groups (both 15%, p = 0.99). Newly required postoperative IABP support was equivalent between groups (WB: 15% vs. DN: 10%, p = 0.499). Use of postoperative inotropes and dosage used were similar between groups. Similarly, postoperative complications such as, unplanned reoperation, readmission to ICU, sepsis, renal failure, atrial fibrillation, and stroke were not significantly different between the 2 groups. Mean total post-operative PRBC transfusion requirement was significantly lower in DN versus WB patients (WB vs. DN, 2.3 ± 2.4 vs.1.3 ± 1.5, p = 0.033). A trend towards lower duration of ventilation, reduced incidence of postoperative atrial fibrillation, reduced length of ICU stay, and lower length of hospital stay was present in DN versus WB patients.

**Table 4:** **Post-operative outcomes**

	WB	dN	***p***
n	40	40	
Postoperative inotropic support	26 (65%)	28 (70%)	0.152
Number of inotropes	1.9 ± 1	2.0 ± 0.9	0.449
Dobutamine dose (μ/kg/min)	4.2 ± 1.1	6.0 ± 1.4	0.124
Norepinephrine dose (μ/min)	4.0 ± 2.6	3.6 ± 2.7	0.592
Vasopressin dose (units/hr)	2.4 ± 1.5	2.7 ± 1.5	0.600
Milrinone dose (μ/kg/min)	0.3 ± 0.1	0.3 ± 0.1	0.057
Ventilation duration (hours)	44.7 ± 87	30.6 ± 47	0.373
Total PRBC Transfusion (mean) (units)	2.3 ± 2.4	1.3 ± 1.5	0.033
ICU stay (days)	7.5 ± 14	4.0 ± 3.0	0.133
Postoperative hospital stay (days)	20.2 ± 24	12.4 ± 9.0	0.048

Values are means ± SD, or counts (%). PRBC-packed red blood cells, ICU-intensive care unit.

Independent predictors of LCOS in the matched population are depicted in Table 5. LVEF < 30% carried the highest risk of LCOS (OR 7.5, CI: 1.5-37.4) followed by reoperative cardiac surgery (OR 4.7, CI: 1.2- 45.8) and then preoperative cardiogenic shock (OR 4.2, CI: 1.6-28.9), respectively. Importantly, cardioplegia technique was not found to be an independent predictor of LCOS (OR 0.9, CI: 0.3-2.7). The multivariable model c statistic was 0.34 and the Hosmer-Lemeshow goodness of fit statistic was 0.63. No multicollinearity was identified.

**Table 5:** **Predictors of low cardiac output syndrome (LCOS)**

	Odds Ratio	***p***	95% C.I. for Odds Ratio	
			Lower	Upper
DN cardioplegia	0.879	0.821	0.289	2.675
Female gender	2.056	0.222	0.648	6.526
Preoperative arrhythmia	2.799	0.117	0.773	10.127
Low LVEF, <30%	7.526	0.014	1.515	37.395
Previous Cardiac Surgery	4.735	0.039	1.154	45.809
Shock	4.198	0.029	1.610	28.914
NYHA class, >3	1.187	0.801	0.314	4.492

DN-Del Nido, LVEF-left ventricular ejection fraction, NYHA-New York Heart Association.

## Discussion

Despite advances in anesthesia, surgical techniques, perioperative care, and myocardial protection, low cardiac output syndrome after CABG for AMI confers substantial morbidity and mortality in this population, as high as 4-7% 30-day mortality in some recent series [2],[7]. Numerous myocardial protective solutions have been used after AMI, most notably warm whole blood and Buckberg solutions formulated to replenish ATP stores and essential electrolytes necessary for myocyte contractility [8]. DN solution has not been studied in the AMI setting, and results of its use in the daily practice of adult cardiac surgery have yet to be documented. In this manuscript, we present our experience with Del Nido cardioplegia, a hyperkalemic, low calcium cardioplegic solution with lidocaine and magnesium additives, in patients undergoing CABG for AMI, and compare our outcomes to a propensity-matched cohort of patients who received our standard WB solution in the current era. Our primary findings were that: 1) there was no in-hospital mortality difference in patients receiving DB vs. WB cardioplegia, 2) intraoperative myocardial protection, as evidenced by need for inotropes or circulatory support, was equivalent in DN and WB patients, 3) DN use was associated with shorter CPB and XC times. At our institution, DN has been used for all adult cardiac surgery since its introduction in 2011.

Two distinct advantages of DN over WB cardioplegia are apparent from our study data. First, operations utilizing DN were shorter, despite having similar preoperative risk factors and undergoing similar numbers of bypass grafting, which we attribute to the reduced time required for cardioplegia administration. With DN, the need for retrograde and SVG cardioplegia administration is reduced (although not eliminated depending on coronary anatomy), and the hassle of repeated cardioplegia dosing is eliminated. Repeated dosing can be cumbersome, can interrupt the flow of the operation, and can be difficult to time properly. In this setting, a single-dose agent is highly preferable. Moreover, the reduced ischemic time (cross-clamp) could prove to have beneficial effects on clinical outcomes in a larger sample size.

A lower total volume of cardioplegia may reduce hemodilution while on bypass, thus lowering the requirement for transfusion. The benefits of preventing hemodilution has been shown with prior cardioplegia solutions - standard WB or undiluted blood (microplegia) cardioplegia offer superior myocardial protection when compared with crystalloid cardioplegia [9]. Moreover, microplegia has been shown to improve recovery by limiting myocardial edema [10]. Although the optimal dilution of cardioplegic solutions is still unknown, the added hemoconcentration afforded with DN administration should limit edema further than WB or microplegia. In our data, total blood transfusion requirement was lower with DN versus WB, likely as result of lower total cardioplegia volume. Secondary benefits may potentially include lower duration of ventilator support and reduced hospital stay.

During AMI, a complex series of biochemical and metabolic changes in myocardial tissue occur due to deprivation of oxygen and nutrient supply, causing myocardial tissue to behave differently than other conditions. Consequently mitochondrial damage and energy depletion impair myocardial contractile function [11]. Anaerobic glycolysis due to the absence of oxygen results in the accumulation of lactate and intracellular pH reduction (to <7.0). The latter activates the Na^+^-H^+^ ion exchanger, thus extruding protons from the cell in exchange for Na^+^ entry. The impaired function of (Na + K)-ATPase contributes to exacerbate the intracellular Na^+^ and Ca^2+^ overload, which in turn worsens intolerance to ischemia [3]. Moreover, during hyperkalemic cardioplegic arrest (K^+^ 16-20 mmol/L), membrane potential depolarization occurs at a membrane potential of -35 to -64 mV, at which point a small percentage of Na^+^ and Ca^+2^ channels may continue to be active. The net result is continued intracellular accumulation of Ca^+2^ during arrest, which the cell counteracts through energy requiring active transport mechanisms, and ultimately manifesting as myocardial dysfunction upon reperfusion. It is theorized that the lidocaine content in DN serves to increase Na^+^ channel blockade and minimize the potential for Na^+^ window current [5]. This, in addition to its Mg^2+^ content acting as Ca^2+^ antagonist, may represent an important mechanism of benefit of DN cardioplegia. An effective reduction in intracellular calcium as a result of this mechanism has been demonstrated in animal hearts arrested with DN solution - diastolic intracellular calcium levels were significantly lower in DN hearts compared to standard WB solution, without a negative contractile effect after recovery [12]. Furthermore, a study involving both animals and pediatric patients [5] comparing WB and DN cardiplegia also demonstrated superior calcium handling of rat cardiomyocytes exposed to DN and reduced troponin T release after surgery in pediatric patients. Maintenance of primary calcium handling mechanisms may the most important biologic mechanism of cytoprotection of DN, but requires further investigation.

The limitations inherent to a retrospective analysis are present in this study. Although all patients in our recent experience were included and matched for preoperative risk using a propensity analysis, the possibility of selection bias exists due to differing surgeons, practice referral patterns for CABG, and the evolving management of AMI using PCI with modern stent platforms and pharmacology. Using recent data and consecutive patients should minimize this bias, although cannot be excluded short of a randomized trial. In addition to cohort size being small, some covariates such as intra- and postoperative hemodynamic parameters, completeness of revascularization, cardiac biomarker dynamics in postoperative period, and surgeon's level of experience could not be included in the analysis. Nonetheless, our operative techniques and postoperative management remained fairly similar during the time of these 2 cohorts.

## Conclusion

Our study showed that myocardial protection with DN solution in isolated CABG surgery following AMI was associated with mortality, complications, and preservation comparable to WB cardioplegia. The reduced CPB and XC times with DN solution, as well as lower total volume of cardioplegia, may help shorten lengths of stay and improve clinical outcomes. In addition, the added benefit of maintaining continuity of the operation without the need for repeated cardioplegia doses cannot be overlooked. Our study is the first to show clinical outcomes and benefits of dN cardioplegia in the setting of coronary revascularization for AMI. Further randomized clinical trial study of the clinical benefit of DN with single-dose administration, effect on cytoprotective mechanisms of membrane stabilization, and dosing regimen in this sick population is warranted.

## Authors’ original submitted files for images

Below are the links to the authors’ original submitted files for images. Authors’ original file for figure 1 Authors’ original file for figure 2

## Abbreviations

AMI: Acute myocardial infarction
ATP: Adenosine triphosphate
BMI: Body mass index
CABG: Coronary artery bypass grafting
CI: Confidence intervals
CPB: Cardiopulmonary bypass
DN: Del Nido
ECMO: Extra corporeal membrane oxygenation
IABP: Intra-aortic balloon pump
LCOS: Low cardiac output syndrome
LVEF: Left ventricular ejection fraction
MCS: Mechanical circulatory support
NYHA: New York heart association
OR: Odds ratio
PCI: Percutanous coronary intervention
PRBC: Packed red blood cells
SD: Standard deviation
STS: Society of thoracic surgeons
WB: Whole blood
XC: Cross clamp
