# Supplementary figures and images for: Del Nido Cardioplegia can be safely administered in high-risk coronary artery bypass grafting surgery after acute myocardial infarction: a propensity matched comparison
Source: J Cardiothorac Surg. 2014 Oct 30;9:141. doi: 10.1186/s13019-014-0141-5 (PMC4220058; doi:10.1186/s13019-014-0141-5)

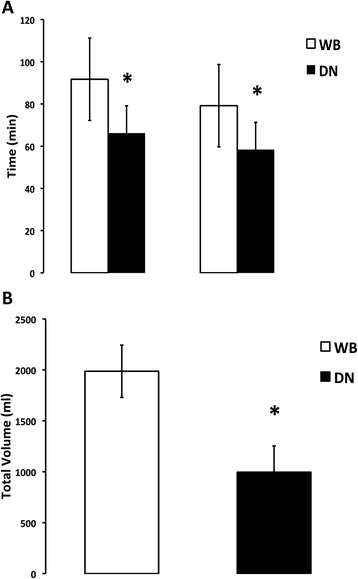

Supplement: Supplementary file 1 — Authors’ original file for figure 1 [file 13019_2014_141_MOESM1_ESM.gif]

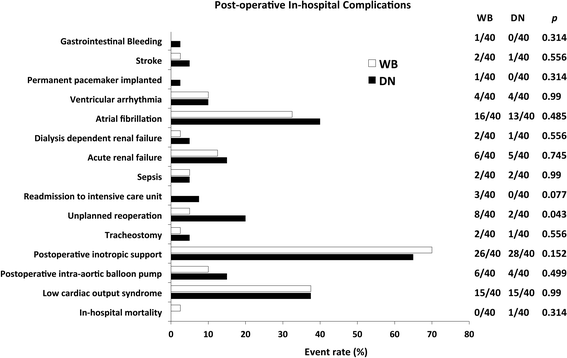

Supplement: Supplementary file 2 — Authors’ original file for figure 2 [file 13019_2014_141_MOESM2_ESM.gif]
